# Three‐Year Diagnostic Delay in Shrinking Lung Syndrome: Rituximab‐Theophylline Combination as Rescue Therapy

**DOI:** 10.1002/rcr2.70461

**Published:** 2026-01-07

**Authors:** Somayeh Motamed, Alireza Mirzamohamadi, Shokufe Sadeghi, Vahid Ardestani Rostami, Mehrdad Mahalleh, Majid Alikhani

**Affiliations:** ^1^ Rheumatology Research Center Tehran University of Medical Sciences Tehran Iran

**Keywords:** case report, rituximab, shrinking lung syndrome, systemic lupus erythematosus, theophylline

## Abstract

Shrinking lung syndrome (SLS) is an infrequent complication of systemic lupus erythematosus (SLE). A 41‐year‐old woman with a 10‐year history of SLE presented with dyspnea, chest pain, and dry cough. She had poor medication compliance and multiple hospitalizations over 3 years for similar symptoms, without a definitive diagnosis. Imaging revealed bilateral basal atelectasis and an elevated right hemidiaphragm, while pulmonary function tests (PFTs) showed a restrictive pattern. Other lung and cardiac conditions were excluded, leading to a diagnosis of SLS. Treatment with rituximab, prednisolone, and theophylline improved PFTs, chest pain, cough, and dyspnea at follow‐up. Clinicians should consider SLS in SLE patients with dyspnea and characteristic imaging, as delayed diagnosis may increase morbidity.

## Graphical abstract

This report presents a case of systemic lupus erythematosus with multiple hospitalizations over 3 years due to unexplained respiratory symptoms (dyspnea, reduced lung volume, elevated diaphragm, coughing, and chest pain), ultimately diagnosed with shrinking lung syndrome (a rare complication of lupus without established treatment) and successfully treated with rituximab, prednisolone, and theophylline.

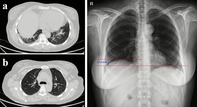

## 1 Introduction

Shrinking lung syndrome (SLS) is a rare complication of autoimmune diseases, especially systemic lupus erythematosus (SLE), occurring in about 1% of patients after 4–6.5 years [1, 2]. Symptoms include dyspnea, reduced lung volume, elevated diaphragm, coughing, and chest pain [2]. Its possible causes include diaphragmatic weakness, abnormal chest wall expansion, pleural inflammation, and phrenic neuropathy [3]. Due to its rarity and nonspecific symptoms, SLS is often overlooked with significant diagnostic delays (mean 11 ± 20 months, with 48% of cases diagnosed after > 1 year of symptoms) and can be mistaken for conditions like pleuritis or pulmonary embolism [2]. Although there is no evidence‐based guideline for treatment, previous reports suggest corticosteroids, inhaled β‐2 agonists, theophylline, azathioprine, cyclophosphamide, analgesics, and physiotherapy [2]. Recent studies have shown that rituximab is emerging as a promising option in resistant cases, with case reports indicating success after initial steroid treatment fails [4]. However, further research is warranted to confirm its effectiveness.

Herein, we present a case of SLE with multiple hospitalizations over 3 years due to unexplained respiratory symptoms, ultimately diagnosed with SLS and treated with rituximab, prednisolone, and theophylline.

## 2 Case Report

In 2023, a 41‐year‐old Azeri woman with a 10‐year history of SLE was referred to our clinic. The diagnosis was established according to the 2019 European League Against Rheumatism/American College of Rheumatology Classification (EULAR/ACR) criteria, supported by the following findings: polyarthritis, lupus nephritis (Class III), positive fluorescent antinuclear antibody (FANA) with a speckled pattern (titre = 1/80), low complement levels (C3 and C4), and a positive anti‐double‐stranded dNA (ds‐DNA) antibody. She presented with recurrent dyspnea (Modified Medical Research Council (mMRC) grade 2), pleuritic chest pain, and dry cough. These symptoms began in 2020 and led to multiple hospitalizations without a definitive diagnosis for 3 years. The patient reported poor medication adherence due to side effects, especially weight gain, and a general reluctance to take pills.

On examination, vital signs were stable: blood pressure 130/70 mmHg, pulse 90 bpm, temperature 36.5°C, oxygen saturation 95% at rest (on room air) and 93% during exertion. Lung auscultation was normal, and there were no skin rashes, oral ulcers, or arthritis. Her height was 162 cm, and her weight was 67 kg (body mass index (BMI) = 25.5 kg/m^2^).

Paraclinical tests (Table 1) revealed significantly elevated levels of autoantibodies, low complement components, and proteinuria (902 mg in 24 h). The patient's urine analysis showed evidence of urinary tract infection (bacteriuria, leukocyturia, and positive *E. coli* culture), which explains the elevated CRP.

**TABLE 1:** Autoantibodies, complement components, and inflammatory markers results.

Test	Patient value	Normal range
ANA (IU/mL)	100	< 12
RF (IU/mL)	28.2	≤ 20
Anti‐dsDNA (IU/mL)	> 200	< 20
Anti‐SSA (U/mL)	> 200	≤ 25
Anti‐SSB (U/mL)	> 200	≤ 25
Anti‐CL IgM (MPL U/mL)	> 80	≤ 7
Anti‐B2GPI IgM (U/mL)	59.8	≤ 8
C3 (mg/dL)	63	90–180
C4 (mg/dL)	10	10–40
ESR (mm/h)	88	0–25
CRP (mg/L)	24.0	≤ 6.0

Abbreviations: ANA, antinuclear antibody; Anti‐CL IgM, anti‐cardiolipin immunoglobulin M; Anti‐dsDNA, anti‐double‐stranded DNA; Anti‐SSA, anti‐Sjögren's syndrome‐related antigen A; Anti‐SSB, anti‐Sjögren's syndrome‐related antigen B; Anti‐B2GPI IgM, anti‐beta‐2 glycoprotein I immunoglobulin M; C3, complement component 3; C4, complement component 4; CRP, C‐reactive protein; ESR, erythrocyte sedimentation rate; RF, rheumatoid factor.

**FIGURE 1:**
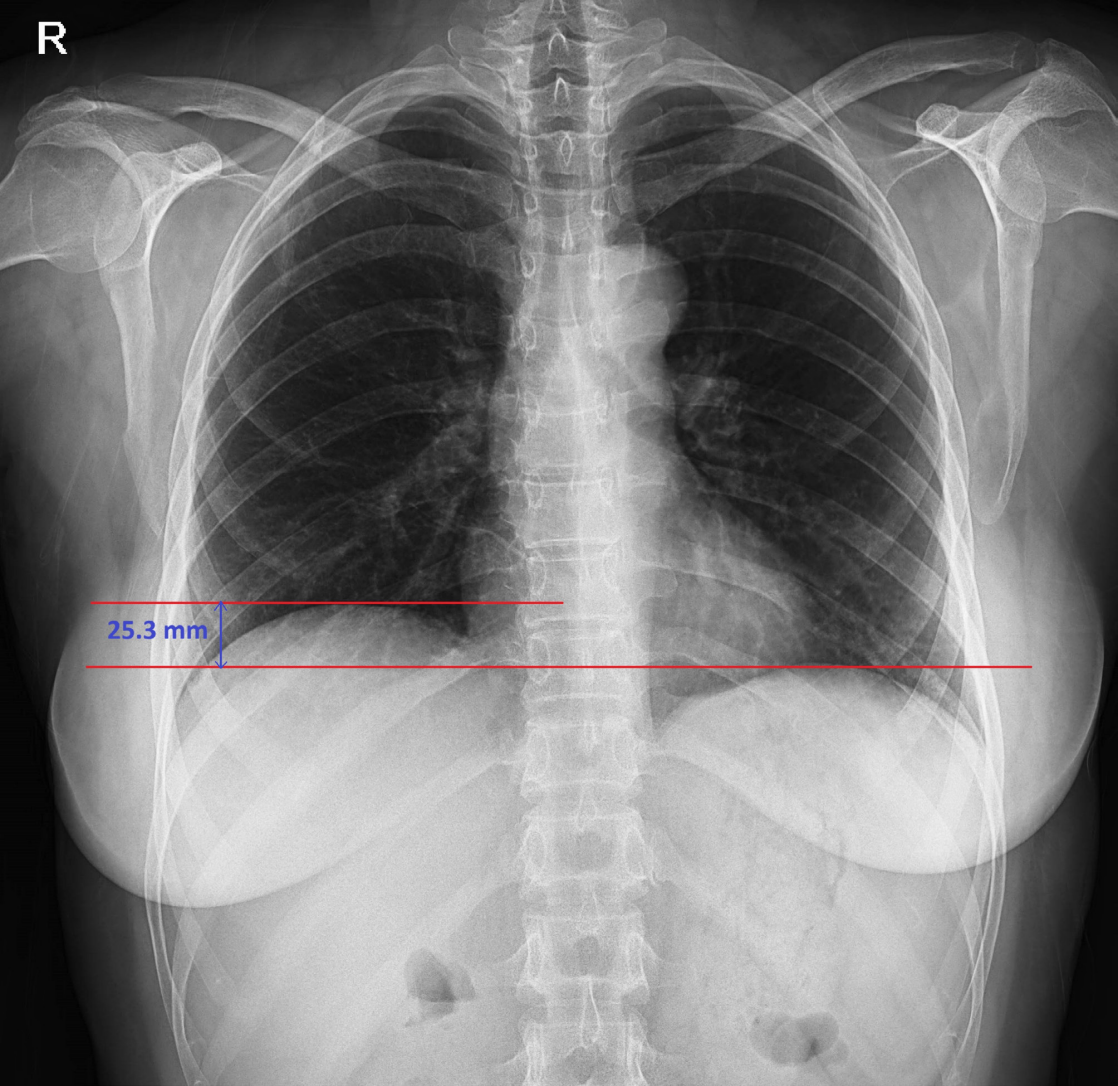
Chest radiograph demonstrating elevated right hemidiaphragm (25.3 mm measurement shown with calliper).

**FIGURE 2:**
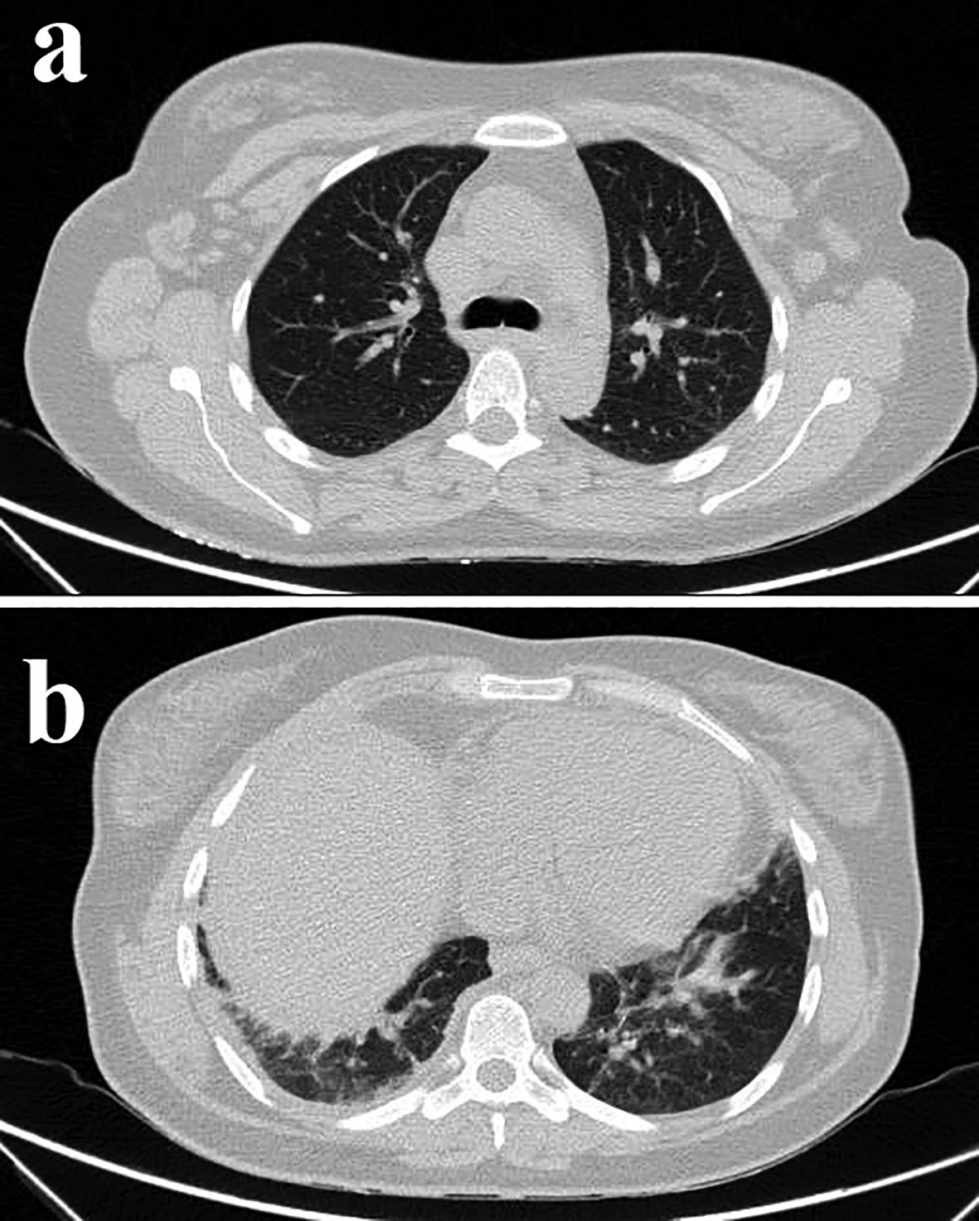
Thoracic spiral CT scan findings: (a) No evidence of active parenchymal lung disease; (b) Bilateral basal atelectasis. Note the absence of pleural or pericardial effusions.

The results of pulmonary function tests (PFTs) showed a restrictive pattern (Table 2: 2023 column). Subsequently, the chest X‐ray revealed an elevated right hemidiaphragm (Figure 1). The spiral chest CT scan revealed bilateral atelectasis predominantly at the lung bases (Figure 2b) without active parenchymal lung disease, vascular pathology, or pleural/pericardial effusion (Figure 2a). However, there was evidence of splenomegaly and mild perihepatic fluid.

**TABLE 2:** Patient's pulmonary function test results (2020: Onset of respiratory symptoms, 2023: Diagnosis and initiation of treatment, 2025: Most recent follow‐up).

Pulmonary function test	Unit	Pred			Act			Act/pred		
		2020	2023	2025	2020	2023	2025	2020	2023	2025
FVCex	l	3.28	3.2	3.11	2.63	2.1	2.74	80%	67%	88%
FEV1	l	2.84	2.7	2.67	2.21	1.8	2.28	78%	66%	85%
FEV1/FVCex	%	82%	81%	81%	84%	85%	83%	102%	105%	102%
MEF25‐75	l/S	3.68	3.5	3.49	2.62	2.44	2.73	71%	69%	78%
AREAex	l*l/S	8.95	8.6	—	7.41	5.33	—	83%	62%	—
RAW	kPas/l	< 0.35	< 0.35	—	0.33	0.54	—	95%	155%	—
TLC‐B	l	4.84	4.9	—	4.2	3.55	—	87%	72%	—
RV‐B	l	1.51	1.59	—	1.72	1.44	—	114%	90%	—

Abbreviations: FEV1: forced expiratory volume in the first second of forceful expiration; FVC: forced vital capacity; MEF: mean expiratory flow; RAW: specific airway resistance; RV: residual volume; TLC: total lung capacity.

The pelvic and abdominal ultrasonography was normal except for splenomegaly (diameter of 140 mm) with a homogeneous echotexture of parenchym.

To rule out pulmonary thromboembolism, CT pulmonary angiography, a ventilation/perfusion scan, and a D‐dimer test were done, which were all normal. Furthermore, bilateral lower limb venous colour Doppler ultrasound showed no evidence of deep vein thrombosis. Echocardiography results were normal, excluding cardiac causes of dyspnea.

Based on the Systemic Lupus Erythematosus Disease Activity Index 2000 (SLEDAI‐2 K) assessment, the patient's disease activity score was 8 (considered as moderate activity), attributed to proteinuria (4 points), low complement levels (2 points), and elevated anti‐dsDNA antibodies (2 points).

Ultimately, due to the lack of involvement of other organs causing dyspnea (including the heart, lungs, and nervous system), and given the presence of an elevated diaphragm, the restrictive pattern on PFTs, and SLE disease activity, the patient was diagnosed with SLS in the context of active SLE.

Considering the patient's history of poor medication adherence and disease activity, rituximab (1 g at weeks 0 and 2 as induction therapy, followed by maintenance doses every 6 months) and theophylline (200 mg/day) were also prescribed in addition to prednisolone (30 mg/day). Theophylline was administered for its triple action—bronchodilation, improved diaphragmatic strength, and anti‐inflammatory effects—to complement the immunosuppression provided by corticosteroid and rituximab.

At the six‐month follow‐up, the patient reported significant improvement in dyspnea (mMRC grade 0) and resolution of chest pain and cough. In 2025, repeat PFTs showed normalisation of lung volumes (Table 2, 2025: follow‐up).

## 3 Discussion

SLS is a rare manifestation of SLE primarily affecting women and occurs in the later stages of the disease. Rosenow et al. first identified this condition 50 years ago, describing six SLE patients with respiratory insufficiency caused by decreased lung volume without any pleuro‐parenchymal disease. Notably, the aetiology remains unknown [4].

SLS can develop at any time, ranging from 1 month to 35 years after SLE onset. Although in the present case, SLS occurred during the activity of SLE, the literature indicates that SLS may present at any course of SLE and does not consistently correlate with high global disease activity. Previous studies show that SLS frequently occurs in patients without prior major organ involvement, and more than half of the patients have inactive lupus at the time of diagnosis [2]. Therefore, SLS should be considered in any SLE patient presenting with unexplained dyspnea and diaphragm elevation, irrespective of overall disease activity.

A notable delay in diagnosis is frequently seen in clinical practice, highlighting that SLS remains an under‐recognised pulmonary complication of SLE [2]. To diagnose SLS, other conditions such as pneumonia, viral or bacterial pleurisy, pericarditis, and pulmonary embolism must be ruled out [3]. PFTs help diagnose SLS, showing decreased forced expiratory volume (FEV1) and forced vital capacity (FVC), while the ratio of them (FEV1/FVC) is normal or elevated [2]. Initial recommended paraclinical studies include chest x‐ray and thoracic HRCT, along with pulmonary and diaphragmatic function assessments. Key findings include elevated hemidiaphragms (unilateral or bilateral) with reduced lung volumes, in the absence of parenchymal lung disease or vascular abnormalities [2].

SLS has no definitive cure, but treatments mainly include corticosteroids (prednisolone at a dose of 0.5 mg‐1.0 mg/kg) and other immunosuppressive drugs [4]. Although steroid therapy provides a good prognosis for SLS patients, improves their PFTs, and palliates their symptoms, achieving full recovery from some abnormalities, such as clinical, functional, and radiographic abnormalities, is not common [5]. There is some evidence supporting the potential positive effect of azathioprine, rituximab, cyclophosphamide, methotrexate, theophylline, and β‐2 agonists in relieving symptoms [1].

In previous studies, initial treatment typically involves medium to high‐dose glucocorticoids. For patients with severe decline or insufficient response to steroids, adding an immunosuppressant like azathioprine or cyclophosphamide was recommended. In severe or refractory cases, rituximab has shown effectiveness and safety in limited reports, potentially positioning it as a first‐choice immunosuppressant. Therapies aimed at increasing diaphragmatic strength (such as theophylline or β‐2 agonists), alone or in combination with glucocorticoids, have also been suggested. However, due to the rarity of SLS, there are no randomised trials on the efficacy of these treatments [2].

In managing refractory SLS patients with poor medication adherence, as in our case, we propose the theophylline‐rituximab‐prednisolone combination as a viable therapy. However, to our knowledge, no study has reported using only this combination, and further research is needed to assess its effectiveness. Clinicians should consider SLS when SLE patients have dyspnea with imaging showing an elevated diaphragm and basal atelectasis.

## Author Contributions

A.M.: writing – review and editing, data collection. S.M.: writing – original draft, data collection. M.M.: writing – review and editing. S.S., V.A.: investigation, data collection. M.A.: supervision. All authors have read and approved the final manuscript.

## Funding

The authors have nothing to report.

## Consent

The authors declare that written informed consent was obtained for the publication of this manuscript and accompanying images using the consent form provided by the Journal.

## Conflicts of Interest

The authors declare no conflicts of interest.

## Data Availability

The data that support the findings of this study are available from the corresponding author upon reasonable request.
